# Using the best available data to estimate the cost of antimicrobial resistance: a systematic review

**DOI:** 10.1186/s13756-019-0472-z

**Published:** 2019-02-01

**Authors:** Teresa M. Wozniak, Louise Barnsbee, Xing J. Lee, Rosana E. Pacella

**Affiliations:** 1 Centre for Research Excellence in Reducing Healthcare Associated Infections, Queensland University of Technology (QUT), Brisbane, QLD Australia; 2 Institute of Health and Biomedical Innovation, Queensland University of Technology (QUT), Brisbane, QLD Australia; 3 Australian Centre for Health Services Innovation, Queensland University of Technology (QUT), Brisbane, QLD Australia; 4 University of Chichester, West Sussex, UK; 5 Menzies School of Health Research, Royal Darwin Hospital, Rocklands Drive, Tiwi, Darwin, Northern Territory 0810 Australia

**Keywords:** Antimicrobial resistance, Review, Costs, Hospital, Study design, Framework

## Abstract

**Background:**

Valuation of the economic cost of antimicrobial resistance (AMR) is important for decision making and should be estimated accurately. Highly variable or erroneous estimates may alarm policy makers and hospital administrators to act, but they also create confusion as to what the most reliable estimates are and how these should be assessed. This study aimed to assess the quality of methods used in studies that quantify the costs of AMR and to determine the best available evidence of the incremental cost of these infections.

**Methods:**

In this systematic review, we searched PubMed, Embase, Cinahl, Cochrane databases and grey literature sources published between January 2012 and October 2016. Articles reporting the additional burden of *Enterococcus spp.*, *Escherichia coli (E. coli)*, *Klebsiella pneumoniae (K. pneumoniae)*, *Pseudomonas aeruginosa (P. aeruginosa)* and *Staphylococcus aureus* (*S. aureus*) resistant versus susceptible infections were sourced. The included studies were broadly classified as reporting oncosts from the healthcare/hospital/hospital charges perspective or societal perspective. Risk of bias was assessed based on three methodological components: (1) adjustment for length of stay prior to infection onset and consideration of time-dependent bias, (2) adjustment for comorbidities or severity of disease, and (3) adjustment for inappropriate antibiotic therapy.

**Results:**

Of 1094 identified studies, we identified 12 peer-reviewed articles and two reports that quantified the economic burden of clinically important resistant infections. Two studies used multi-state modelling to account for the timing of infection minimising the risk of time dependent bias and these were considered to generate the best available cost estimates. Studies report an additional CHF 9473 per extended-spectrum beta-lactamases -resistant *Enterobacteriaceae* bloodstream infections (BSI); additional €3200 per third-generation cephalosporin resistant *Enterobacteriaceae* BSI; and additional €1600 per methicillin-resistant *S. aureus* (MRSA) BSI. The remaining studies either partially adjusted or did not consider the timing of infection in their analysis.

**Conclusions:**

Implementation of AMR policy and decision-making should be guided only by reliable, unbiased estimates of effect size. Generating these estimates requires a thorough understanding of important biases and their impact on measured outcomes. This will ensure that researchers, clinicians, and other key decision makers concerned with increasing public health threat of AMR are accurately guided by the best available evidence.

**Electronic supplementary material:**

The online version of this article (10.1186/s13756-019-0472-z) contains supplementary material, which is available to authorized users.

## Background

Quantifying the burden of antimicrobial resistance (AMR) is challenging and encompasses various methodologies that aim to measure the impact on the patient, their use of the healthcare system and/ or contribution to society [1–4]. Erroneous or unclear estimates of impact can have alarming effects some of which may contribute to greater action but they also create confusion and potentially undermine the fight against AMR. Therefore, assessing the quality of the available studies is essential to equip clinicians and policy-makers with the right tools to ensuring that decisions are based on well-designed studies which generate reliable, detailed and actionable measures [5].

The past decade has seen a large influx of studies reporting the costs of AMR [6] and these have produced highly variable estimates ranging from £3-11billion [7] to US$100 trillion [4]. Inconsistency in economic studies examining the burden of AMR have been reported [6, 8–10] and demonstrate the importance of a thorough analysis of the methodologies used to generate these estimates [11]. As much as 84% of the variance in costs between patients infected with a resistant versus susceptible infection can be attributed to factors that could have been adjusted for in the study design [8]. Inadequate adjustment for confounding increases the risk of bias on outcomes, such as cost of infection. Determining the most likely causes of heterogeneity in cost data, therefore may require an analysis of both the clinical differences in participant characteristics as well as variability produced by differences in the methodology and the overall approaches used [12, 13].

Excess length of stay (LOS) is considered the most significant cost of a healthcare associated infection [14]. However, methods to estimate the excess LOS attributable to infection have been shown to be subject to bias, demonstrating one of the key challenges with assessing the costs of infections [15]. Previous studies show that if the timing of infection is not treated as a time-dependent exposure, then bias is introduced resulting in overestimation of excess LOS [15]. As infection is a time-dependent exposure, methods assessing its impact on LOS need to treat it in a time-dependent manner. Study designs which involve matching do not fully account for this time-dependent bias [10], unless day of infection was used in the matching.

Aside from the potential for bias in outcomes such as excess LOS which are then used to estimate costs, further considerations exist. Between studies, cost estimates will differ based on the economic perspective of each study and which exact costs were included. Estimates will differ across countries due to differences in pricing of relevant services and products, and the type of health system in place. The underlying cost data needs to be considered, such as whether the total hospital budget has been divided to cost a bed day, or whether more specifically exact activities to do with infection have been considered.

We report a systematic review of the economic burden of clinically important hospital- and community-acquired infections. This review expands upon a recently published systematic review [16] and previous rapid review [6], thus literature was searched only between 2012 and 2016. We only included studies that compared patients with drug-resistant and drug-susceptible infections. The cost difference between cases and controls therefore represents an estimate of the additional economic burden of resistance. We describe the diverse approaches of studies quantifying the economic burden of AMR, and provide a narrative review of the costs found. This serves to provide an update of current literature. In the process, we highlight recommended methods for addressing bias in estimations.

## Methods

This systematic review is reported according to the PRISMA checklist [17]. Ethical approval was not required for this study. Methods and inclusion criteria were specified in advance and documented in an unregistered review protocol, full text of which is available in Additional file 1. Published articles reporting the economic burden of the following infections: *Enterococcus spp.*, *E. coli, K. pneumoniae, P. aeruginosa* and *S. aureus* compared to susceptible infections, were searched in Pubmed, Embase, Cinahl and Cochrane databases from January 2012 until October 2016. Search strategies are provided in Additional file 1.

### Study selection and data extraction

TW and LB independently screened titles and abstracts of records identified through database searching. Duplicates were removed prior to screening. Full-text articles identified in peer-reviewed and grey literature that met the inclusion criteria were retrieved. Studies were eligible for inclusion if they: reported empirical or primary evidence about the economic impact of resistant versus susceptible infections, or reported about models of this impact; pertained to community or healthcare acquired *Enterococcus spp., Escherichia coli (E. coli)*, *Klebsiella pneumonia (K. pneumoniae)*, *Pseudomonas aeruginosa (P. aeruginosa)*, or *Staphylococcus aureus (S. aureus)*; reported the costs of resistant infections compared to susceptible infections; reported the control group as the susceptible strain of the organism; were published between 2012 and last date of database searching (11th October 2016); were conducted in adult populations (those admitted to adult hospitals). As the review process unfolded, some studies were found to include a mixture of adult and paediatric populations, and these were included as they were considered important studies to describe. We excluded studies that only reported on LOS and/or mortality; studies which compared uninfected patients with patients with resistant infection; studies comparing reduced susceptibility; and, studies focussed on control interventions, cost-effectiveness of interventions or on antiviral, anti-malarial or antiprotozoal infections.

We searched the reference lists of included studies to further identify eligible articles. Reviewers were not masked to the journals or authors of the studies. Data were extracted and condensed into summary tables using Microsoft Word and Excel (Additional file 1).

### Assessment of economic studies

We developed a framework for the assessment of included studies. This framework considered the main parameters of perspective, methodology and the minimum study characteristics reported in each study (Table 1).

**Table 1:** Framework for assessment of economic studies

	Parameters for assessment
Study perspective	What is the study perspective? The study perspective (s) is the viewpoint from which the intervention’s costs and consequences are evaluated [42].
	Patient perspective Example: out-of-pocket costs or patient preference, costs that the patient pays for that are not covered by their health insurance
	Healthcare payer perspective Example: attributable costs to the payers of healthcare including insurers or national payers
	Healthcare system perspective Example: cost effectiveness analysis of stewardship programs, additional length of hospital stay, impact of resistant infections on structuring of cares such as infection control, increased use of isolation rooms
	Societal perspective Example: broader costs to society such as productivity loss due to morbidity or premature death, can include cost to other sectors such as impact on trade and economy
Methodology	Did the study match the resistant cases and susceptible control groups based on LOS prior to infection? or
	Did the study adjust statistically for prior LOS? or
	Did the study conduct sensitivity analysis that considered hospital LOS prior to infection? or
	Was multistate modelling used to take into account the time-varying nature of infections?
	If yes, low to moderate risk of time dependent bias
	If no, high risk
	If unsure, check: Did the study estimate excess LOS, mortality or costs without matching by LOS prior to infection
	Did the study express only post-infection LOS or costs?
	If yes to any of the above then high risk of time-dependent bias
	Did the study adjust for underlying co-morbidities or severity of illness on clinical outcomes
	If yes, did they adjust for time-dependent bias (above)?
	If no, high-risk of bias
	Did the study adjust for inappropriate antibiotic therapy
	If yes, did they adjust for time-dependent bias (above)?
	If no, high-risk of bias
Minimum study characteristics to be reported	Country
	Year of data used for analysis
	Organism, susceptibility, and site of infection
	Comparator
	Study design and analysis methods
	Cost driver/ costs explored (e.g., excess LOS, mortality)
	Type of costs (including year of cost data and currency)
	Statistical significance

### Risk of bias assessment

To assess the quality of included studies, we developed a risk of bias assessment tool modified from previously identified key methodological caveats of costing studies [10] (Additional file 2). This tool assessed included studies against three methodological components: (1) adjustment for LOS prior to infection onset and consideration of time-dependent bias, (2) adjustment for comorbidities or severity of disease, and (3) adjustment for inappropriate antibiotic therapy. In the adjustment for LOS and disease severity, we also assessed whether the adjustment was made at the study design phase (i.e. matching on LOS or underlying comorbidities) or at the analysis phase (i.e. multi-state modelling or adjusting for time to infection as a baseline covariate in regression analysis). Matched study designs were not considered to fully address time-dependent bias, as has been previously shown [15]. Inappropriate empiric therapy was considered due to its potential influence on patient outcomes [18].

### Extraction of cost estimates

From the included studies, we extracted cost estimates, the year of cost data and cost drivers. Usually, study perspectives in economic evaluations are those of the patient, hospital/clinic, healthcare system or society [19]. Depending on the country and context, the specific payer and costs included may vary. For example, in one study, the cost perspective is described as that of the hospital, and this involved a third-party payer perspective [20]. Further, physician remuneration costs were not included as they were not reimbursed through the hospital, but fell under a particular insurance plan [20]. As our purposes were descriptive in nature, rather than to compare in detail the costs included in each study, we did not exclude by study perspectives, and extracted costs, charges or estimates as presented by the studies. We broadly described the perspectives as either healthcare system/hospital/hospital charges to patients or as societal. Interested readers may refer to the individual studies to find exact perspective and cost/charge descriptions.

Costs and charges reported in the included studies were not converted to a single currency for comparative purposes. Due to differences in healthcare systems and pricing of healthcare items in different countries, it was considered that conversion could be misleading, because the cost to treat an infection in one setting would likely be different in another. For studies that did not report hospital LOS and cost of resistance, we subtracted the LOS or cost estimate of susceptible infections from resistant infections to provide an overall estimate of effect size.

### Synthesis

Heterogeneity of studies did not permit for a meta-analysis of combined effect.

## Results

**Fig. 1:**
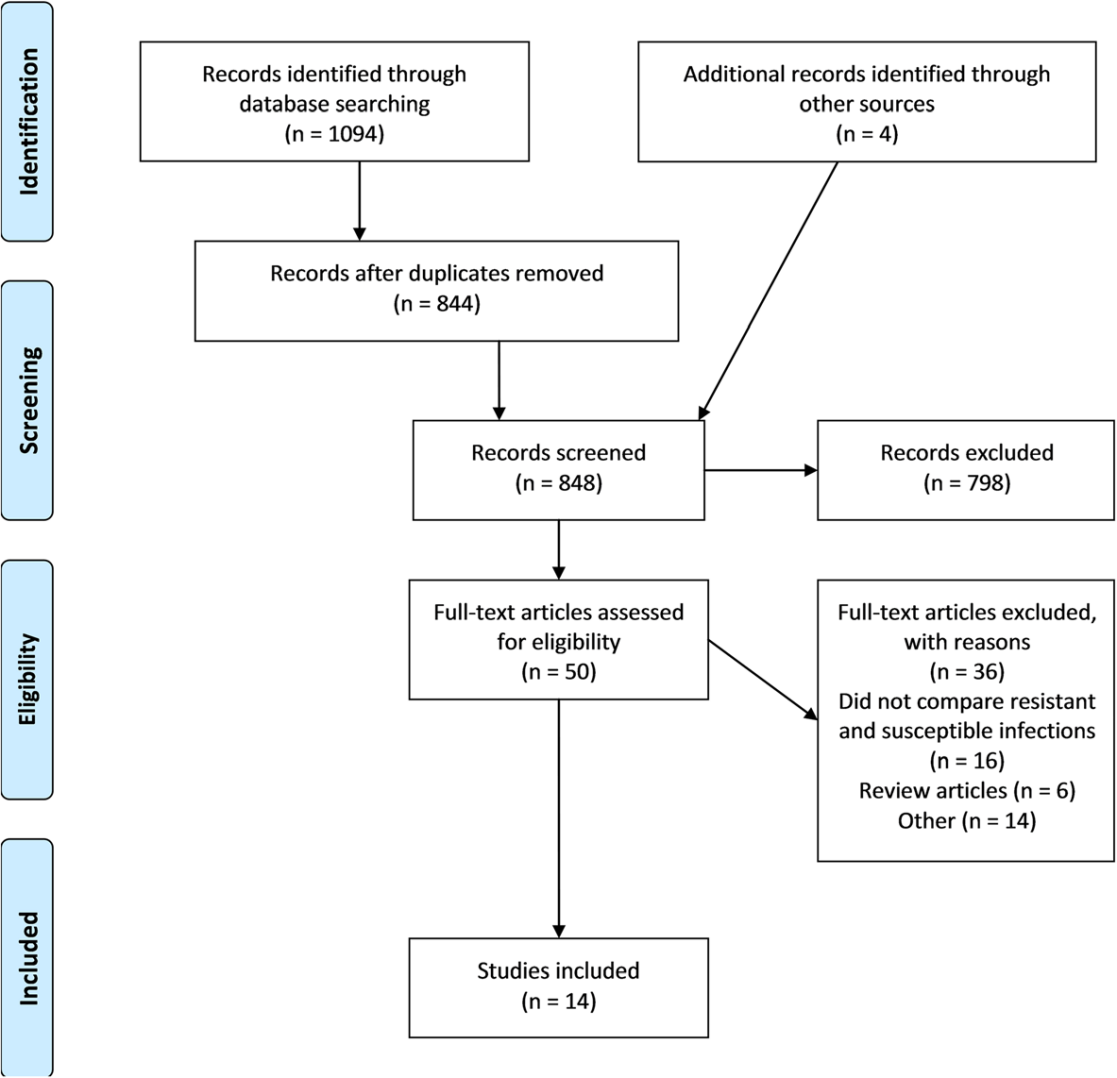
PRISMA flow diagram of the search

Database searching returned 1094 articles, of which 844 articles were extracted. A further three reports [4, 21, 22] were identified from grey literature searching. Hand searching identified one relevant article [23]. Screening based on title and abstract excluded 798 records. Of 50 articles assessed for eligibility, 14 studies met the inclusion criteria (Fig. 1) [20–33]. Reasons for full-text exclusion are given in Fig. 1. “Other” included reasons such as article not in English, poster and oral abstracts that did not provide adequate information, and incorrect patient group.

There were seven cohort studies [20, 23–25, 30, 31, 33]; two case-control studies [28, 29]; two retrospective analyses [26, 27]; one descriptive study [32] and two economic model reports [21, 22]. Studies reported on clinical isolates from BSI, urinary tract infections and other clinical sites (Table 2).

**Table 2:** Study characteristics, graded by risk-of-bias tool with highest quality studies reported at the top of table. 2012–2016

Author (year) Country Year of data used	Organism Site of infection	Comparator (n)	Adjustment for prior LOS or time dependence	Adjustment for disease severity	Adjustment for inappropriate antibiotic use
Stewardson (2016) 10 EU countries 2010–2011 [24]	Enterobacteriaceae *S. auerus* BSI	MSSA (885) MRSA(163) 3GCSE(2100) 3GCRE (360)	Fully adjusted for at the analysis stage using multi-state modelling	Comorbid conditionscalculated and adjusted in model	No
Stewardson (2013) Switzerland 2009 [25]	Enterobacteriaceae BSI	Non-ESBL (96) ESBL+ (30)	Fully adjusted for at the analysis stage using multi-state modelling	Data collected but not adjusted in model	Uncertain if adjusted in model
Neidell (2012) USA 2006–2008 [26]	*S. aureus*, Enterococcus, KP, PA BSI, UTI, Lower RTI	Susceptible (3880) Resistant (1819)	Partially adjusted for at the analysis stage using nearest neighbour matching, based on propensity scores for prior LOS	CCI calculated; individual comorbidities adjusted in model	No
Campbell (2013) USA 2005–2010 [33]	*S. aureus* Multiple sites^a^	MSSA (206) MRSA (119)	Partially adjusted for at the analysis stage by adjusting for time to infection as baseline covariate. In sensitivity analysis matching based on propensity scores for time to infection	CCI calculated and adjusted in model	No
Leistner (2014) Germany 2008–2010 [29]	*E. coli* BSI	Non-ESBL (92) ESBL+ (92)	Partially adjusted for at the design stage using matching (LOS of controls matched with LOS of cases)	Matched on CCI	No
Cheah (2013) Australia 2002–2010 [31]	Enterococcus BSI	VSE (603) VRE (116)	Partially adjusted at the analysis stage using LOS prior to infection	CCI calculated and adjusted in model	Yes
Morales (2012) Spain 2005–2006 [27]	*P. aeruginosa* Multiple^b^	Susceptible (149) Resistant (119) MDR (134)	Not addressed	No	No
Maslikowska (2016) Canada 2010–2013 [28]	*E. coli* *Klebsiella* spp. Multiple ^c^	Non-ESBL (75) ESBL+ (75)	Not addressed	No	No
Thampi (2015) Canada 2007–2010 [20]	*S. aureus* BSI	MSSA (377) MRSA (58)	Not addressed	No	No
EstevePalau (2015) Spain 2010–2013 [30]	*E. coli* UTI	Non-ESBL (60) ESBL+ (60)	Not addressed	No	No
MacVane (2014) USA 2011–2012 [23]	*E. coli* *Klebsiella* spp. UTI	Non-ESBL(55) ESBL+ (55)	Not addressed	No	No
Chandy (2014) India 2010 [32]	All organisms BSI suspected	Susceptible (87) Resistant (133)	Not addressed	No	No

*BSI* Bloodstream infection, *UTI* Urinary tract infection, *RTI* Respiratory tract infection, *3GC* Third-generation cephalosporin, *3GCSE* 3GC susceptible *Enterobacteriaceae*, *3GCRE* 3GC resistant *Enterobacteriaceae*, *ESBL* Extended-spectrum beta lactamase, *VSE* Vancomycin susceptible *Enterococcus*, *VRE* Vancomycin resistant *Enterococcus*, *MRSA* Methicillin *resistant S. aureus*, *MSSA* Methicillin susceptible *S.aureus*, *KP K. pneumoniae*, *PA P. aeruginosa*, *MDR* Multidrug resistant, *CCI* Charlson comborbidity index, *APACHE* Acute physiology and chronic health evaluation, *DRG* Disease-related group, *NS* Not significant

^a^multiple sites of infections were, as listed by authors, “blood, urine, respiratory, neurologic, orthopaedic, other”

^b^Respiratory, SST, genitourinary, catheter, endovascular, abscess, peritonitis, digestive, as found in original article

^c^multiple sites of infection are summarised here as orthopaedic, lung, blood, urinary tract, abdominal region, and skin and soft tissue infections

AMR was defined as either (i) the presence or absence of resistance to a given antibiotic with or without intermediate threshold isolates [24, 25, 33]; or (ii) a change in the minimum-inhibitory concentration relative to the baseline levels [27, 29]; or (iii) determination of resistant genotypes [31] or (iv) a combination of microbiologic results, clinical symptoms and diagnosis codes as judged by an expert panel [26]. Standard methods conforming to Clinical Laboratory Standards Institute [20, 28, 32] or European Committee on Antimicrobial Susceptibility Testing guidelines [30] were reported.

Twelve studies reported the economic burden of AMR from a healthcare perspective, with the highest quality studies as assessed by the risk-of-bias tool (Additional file 2) listed at the top of the table (Table 2). Two studies used multi-state modelling to fully adjust the timing of infection [24, 25] and they were assessed to generate cost outcomes of highest quality (Table 2). A further four of the included studies made partial adjustment for time of infection, either at the study design stage by matching for prior LOS [29] or at the statistical stage [26, 31, 33], these were graded next best. The remaining six studies did not report adjustment of the study population for prior LOS or consider time-dependent bias, either at the study design or statistical stage of analysis [20, 23, 27, 28, 30, 32] and were therefore graded as generating cost estimates of low-quality. Some of these studies included post-infection costs only [27, 28] and some admitted patients to the study post-infection only, in an attempt to reduce confounding [20].

Five of the included studies adjusted for severity of disease, primarily using tools such as the Charlson comorbidity index (CCI) and the Acute Physiology and Chronic Health Evaluation score [24, 26, 29, 31, 33] (Table 2). Four of these studies made adjustment for comorbidities at the statistical stage of the analysis and one study matched patients based on CCI [29].

Two studies considered inappropriate initial antimicrobial therapy [25, 31]. Stewardson et al (2013) define this as failure to prescribe an antibiotic that was appropriate for the treatment of a BSI and to which the infecting organism was susceptible within 24 h of the infection [25]. However, it was unclear whether this variable was included in the model. The study by Cheah et al (2013) adjusted for appropriateness of antibiotic therapy in their multivariable model [31]. Neidell et al. [26] adjusts for days of medication used but the adequacy of prior antibiotic use was not considered. One study included patients that were receiving empiric antibiotic therapy [32].

Three studies undertook multivariable regression analysis to determine predictors of increased hospital cost. For resistant *P. aeruginosa* infection, the total hospital cost was 1.37-fold higher (95% CI 1.08–1.72, *p* = 0.01) and 1.77-fold higher (1.41–2.22, *p* < 0.001) for patients infected with a multidrug-resistant strain, compared to non-resistant control [27]. Patients with a MRSA BSI had a 1.32 higher (0.94–1.80, *p* = 0.10) direct costs compared with susceptible *S. aureus* [20]. The impact of extended-spectrum beta-lactamases (ESBL)-producing *E. coli* on hospital costs was increased 3.1-fold (1.3–7.0, *p* = 0.008) compared to non-ESBL *E. coli* urinary isolates [30].

Twelve studies [20, 23–33] and one report [21] quantified the additional LOS of patients with resistant compared to susceptible organisms (Tables 3 and 4). Additional to bed-day estimations, seven of the included studies also reported consumable items used in the clinical management of the infection [20, 23, 25, 27–30]. Not all studies reported the cost of a bed-day as a separate variable, but rather absorbed all of the measured costs into a total estimated burden on the healthcare system. One study reported the economic burden as accounting and opportunity costs [24].

**Table 3:** Attributable LOS and costs associated with AMR infections from a healthcare system/hospital/charges to patients perspective, 2012–2016

Author (year)	Extra LOS due to resistant infection, 95% CI and *P* value (if indicated)	Cost drivers/costs explored	Type of costs (year of cost data) Excess cost, significance (if indicated)
Stewardson (2016) [24]	MRSA: + 2.54 (− 3.19 to 8.27) Third-generation cephalosporin-resistant Enterobacteriaeacea: + 4.89 days (1.11–8.68)	Bed days	Accounting (2011) +€1600 (− 700 to 5000)/ MRSA + €3200 (1600–6000)/ 3GCRE Opportunity +€120 (− 60 to 740)/ MRSA infection +€250 (60–1100)/ 3GCRE infection
Stewardson (2013) [25]	ESBL+ Enterobacteriaceae: + 6.8 days	Bed days Patient activities Hospital service	Accounting (2009) + CHF 9473/ BSI + CHF 284190 total cost of Enterobacteriaceae
Neidell (2012) [26]	Resistant Enterococcus: + 0.85 days (− 0.86 to 2.55) Resistant *K. pneumoniae:* + 1.63 days (− 0.96 to 4.21) Resistant *P. aeruginosa:* + 3.30 days (0.87–5.73), p = < 0.01 Resistant S.*aureus*: + 0.42 days (− 2.29 to 3.13)	Bed Days Medications Procedures	Accounting (*missing*) Enterococcus: +US$16900 (4600–29,300) *p* < 0.01 KP: +US$13200 (− 5900 to 32,200) NS PA:+US$31400 (10100–52,800) p < 0.01 *S. aureus*: -US$16000 (− 36,900 to 4800) NS Results of censored models for HAIs provided.
Campbell (2013) [33]	MRSA: + 5.9 days, *p* = < 0.0001	Bed days Laboratory Pharmacy	Accounting (2009) +US$31338 total from index admission, *p* < 0.05 Results of primary analysis provided.
Leistner (2014) [29]	ESBL+ *E. coli*: − 2 days, *p* = 0.29	Bed days Medical products Laboratory Pharmacy Staff time Procedures	Accounting (*missing*) +€38 / patient per day *p* = 0.69 +€ 1318, *p* = 0.33 (ICU) +€ 930, *p* = 0.7 (general ward) - €1479 total hospital cost, *p* = 0.36
Cheah (2013) [31]	VRE:+ 4.89 days (0.56–11.52)	Bed days	Accounting (2010) + AUD $28,872 (734–70,667)/ hospitalisation
Morales (2012) [27]	Resistant *P. aeruginosa*: + 13.9 days MDR *P. aeruginosa*: + 20.6 days, *p* < 0.00	Bed days Surgery Laboratory Radiology Pathology Pharmacy	Accounting (*missing*) Resistant versus non-resistant: +€7418 MDR versus non-resistant: +€10,332 MDR versus resistant: +€2914/ admission *p* < 0.00 Resistant versus non-resistant OR = 1.37 (95% CI; 1.08–1.72) *p* = 0.01 MDR versus non-resistant OR 1.77 (95% CI; 1.41–2.22) *p* < 0.00
Maslikowska (2016) [28]	ESBL+(*E. coli* + *Klebsiella* spp):+ 2 days, *p* = 0.024	Bed days Other costs^b^	Accounting (*missing*) Canadian dollar +CAD$2625/ admission *p* = 0.039
Thampi (2015) [20]	MRSA:+ 8.5 days, *p* = 0.095	Bed days Human resources^a^ Pharmacy Antibiotics Laboratory Diagnostics Operating room	Accounting (2010) Canadian dollar +C$7153/ patient p = 0.029 OR (MRSA) = 1.32 (0.94–1.8), *p* = 0.1
Esteve-Palau (2015) [30]	ESBL+ *E. coli*: + 4.1 days, *p* = 0.02	Bed days OPAT Pharmacy Antibiotics Laboratory Consultations	Accounting (*missing*) +€1109/ hospitalisation *p* = 0.01 +€2368/ UTI *p* < 0.00 +€1389/ OPAT *p* = 0.04 ESBL versus non-ESBL *E. coli* OR = 3.1 (1.3–7.0) *p* = 0.01
MacVane (2014) [23]	ESBL+(*E. coli* + *Klebsiella* spp):+ 2 days, *p* = 0.02	Bed days Antibiotics	Accounting (*missing*) +US$3189 hospitalisation cost (direct and indirect)/patient (Median loss per patient with ESBL-producing organism versus non-ESBL producing organism.)
Chandy (2014) [32]	Resistant (all) organisms:+ 3 days, *p* = 0.03	Bed days Antibiotics Pharmacy Ward costs	Accounting (*missing*) Indian rupee +INR 41993 (16667–63,848) /hospitalisation, *p* = 0.00

*ESBL* Extended-spectrum beta-lactamases, *MDR* Multidrug resistant, *NS* Not significant, *LOS* Length of stay, *OPAT* Outpatient parenteral antimicrobial therapy

^a^included costs related to nursing and specialists care

^b^included costs, as listed by authors, related to “allied health, ambulatory care, cardiac catheterization, imaging, food, intensive care, laboratory tests, surgical procedures, pharmacy, ward care, and indirect care”

**Table 4:** Studies reporting the economic burden of antimicrobial resistance from a societal perspective, 2012–2016

Author (year) Country	Organism Comparators Site of infection	Methodology	Excess LOS (days)	Cost drivers	Type of costs (year of cost data) Currency Excess cost, significance
Taylor (2010) Global 2010 [21]	*S. aureus*, *E. coli*, *K. pneumoniae*, HIV, malaria, TB MRSA 3GC-*E.coli* 3GC-*K. pneumoniae* Resistant HIV Resistant malaria MDR-TB BSI, UTI, Lower RTI, SSTI	Theoretical dynamic general equilibrium was used to predict future scenarios of incidence and resistance (0%, current rates, 5, 40, 100% resistance) starting with the population in 2010 and projecting to 2050. Costs: (a) increased mortality (b) increased morbidity due to prolonged period of sickness leading to productivity loss Assumptions: (i) Resistance rates increase in a one-off step, not an S-shaped epidemic pattern (ii) Incidence remains constant until 2050 (except malaria where it was projected) (iii) Extra LOS was assumed to be the same for all countries/regions (iv) Mortality risk per infection remained unchanged	Mean excess LOS from the WHO Observatory (2014)	Loss of productivity	Disruption to the supply of labour by increased mortality and morbidity measured as reduction in GDP (2011) US Current cost: US$5.8 trillion Excess cost (over 40 yrs): Loss of US$2.1- $124.5 trillion
KPMG (2014) 156 countries Data sourced from various publication with the latest from year 2012 [22]	*S. aureus*, *E. coli*, *K. pneumoniae*, HIV, malaria, TB Susceptible versus Resistant BSI Lower RTI SSTI UTI	Total factor productivity model used to compute macroeconomic stability, technology, quality of infrastructure, human capital and strength of public institutions. Life expectancy used as a proxy to measure the quality of human capital and adjustments to country life expectancy score were made to allow for deterioration of human capital as result of increased AMR incidence. Labour force was based on working age (15–64) and adjusted to AMR mortality rate Costs: (a) attributable mortality (b) increased morbidity leading to productivity loss. Assumptions: (i) Correction coefficient used to estimate resistance rate by site of infection was assumed to be the same for all countries/ regions (ii) Extra LOS analysed for EU, Iceland and Norway only (iii) Mortality risk per infection remained unchanged	Combined (*S. aureus* + *E. coli* + *K. pneumonia):* 4 mil bed-days in 2012	Loss of productivity + cost of hospital bed-days	Impact on labour force and human capital measured as reduction in GDP (2012) EURO Excess cost: +€1.6 billion Global GDP loss (2050): 40% resistant: 1.66% 100% resistant: 3.4%

*KP K. pneumonia*, *TB Mycobacterium tuberculosis*, *3GC* Third-generation cephalosporin resistant, *BSI* Bloodstream infection, *UTI* Urinary tract infection, *RTI* Respiratory tract infection, *SSTI* Skin and soft tissue infection, *LOS* Length of stay, *GDP* Gross domestic product

The methodology used to generate estimates of cost from a societal perspective were different to those in Table 2, hence these two reports [21, 22] which were identified in grey literature are reported separately (Table 4).

There was inconsistency in the calculation and reporting of bed-days and cost estimates (Tables 3 and 4). Seven studies reported the entire LOS (susceptible or resistant) and associated costs from time of infection [20, 23, 27–30, 33] and the remaining reported the specific excess LOS and the corresponding costs [21, 22, 24–26, 31, 32]. There was a mixture of mean and median cost estimates for a single resistant organism [20, 24–27, 30, 31, 33] or pooled estimates of all resistant organisms [21, 22, 32]. Neidell et al (2012) calculated cost estimates that may capture aspects of societal costs by censoring charges and LOS for patients who died in hospital [26]. While the study’s censored models may capture aspects of societal perspective, for the purpose of this systematic review, this study was grouped with healthcare perspective studies contained in Table 2.

Total hospital cost or charges ranged from additional USD$31,338 for MRSA [33] to CHF284,190 (Swiss Franc) for ESBL-producing *Enterobacteriaceae* [25]. Some costs descriptions were missing and it was unclear whether the costs were total or per hospital admission. Only some studies explicitly stated whether infection control and prevention costs were included. For example, one study explicitly stated infection prevention and control costs were included in the estimation of indirect costs; while another stated they did not estimate the excess cost of isolation of MRSA patients such as costs of additional gowns [20, 28].

By organisms, the costs of resistance were varied. Infection with *Enterobacteriaceae* cost an estimated additional €3200 per third-generation cephalosporin resistant *Enterobacteriaceae* BSI [24] to USD$13,200 per resistant *K. pneumonia*, albeit the latter costs were not statistically significant when compared to susceptible infections [26]. Patients infected with MRSA costs the healthcare system an additional €1600 per BSI [24]; CD$7153 per patient [20] or USD$31,338 per infection [33] compared to methicillin-susceptible *S. aureus*. Vancomycin-resistant *E. faecium* (VRE) costs additional AUD$28,872 per hospitalisation [31] or USD$16,900 from multiple sites of infection [26]. Hospital admissions with resistant *P. aeruginosa* cost an additional €2914 per admission in Spanish hospital [27] and USD$31,400 per infection in a United States hospital [26]. One study estimated the cost of confirmed bacteraemia in patients admitted to a tertiary centre in India to be an additional INR41,993 (Indian rupee) per hospitalisation [32]. Two studies report a negative cost of resistance for ESBL-producing *E. coli* [29] and resistant *S. aureus* [26], albeit both were not statistically significant.

We highlight that high quality estimates arising from appropriate study design and analysis methods are currently available for third-generation cephalosporin-resistant *Enterobacteriaceae* BSI [24]; ESBL-resistant *Enterobacteriaceae* BSI [25] and MRSA BSI [24].

Studies reporting the economic burden from a societal perspective did not employ the same methodologies as healthcare/hospital/hospital charges to patients perspective studies, hence assessment of these was descriptive in nature (Table 4). Both the RAND [21] and KPMG [22] reports present models used to predict future costs incurred to society as a proportion of the loss to the gross domestic product [21, 22]. Reduction in the labour efficiency by means of early death and loss of productivity are also reported. Methodologies of generating costs of LOS or early deaths are less clear in the RAND report. In the absence of data, the authors relied on expert opinion to calculate rates of resistance and/or made assumptions of mortality across regions. The KPMG report presented an additional global estimate of €1.6 billion for *E. coli*, *K. pneumoniae* and MRSA infections [22] and RAND provide a cumulative costs of up to USD$124.5 trillion over a 40 year period for all included organisms (Table 4) [21]. Neither study report confidence intervals but have undertaken sensitivity analysis on key model parameters. The RAND study reports aggregated cost estimates for *E. coli, K. pneumoniae, S. aureus*, HIV, malaria and *M. tuberculosis*, and contribution from individual organisms to the overall cost estimate could not be delineated. Therefore, based on several pitfalls identified in both reports, a definitive estimate of the cost of AMR from the societal perspective could not be derived.

## Discussion

Drug-resistant infections are increasingly prevalent, harder to treat and notoriously challenging to quantify. The most rigorous estimates of the economic impact of AMR are available for third-generation cephalosporin-resistant *Enterobacteriaceae* BSI [24]; ESBL-resistant *Enterobacteriaceae* BSI [25] and MRSA BSI [24] only. The remaining evidence is constructed with methodologies and definitions of exposure and outcomes of varying quality inevitably generating highly variable estimates of the burden of AMR.

### Recommendations to improve estimates of the economic burden of AMR

How exposure (i.e.; AMR) and outcomes (i.e.; additional costs) of interest are defined, measured and reported needs consideration. Published definitions of AMR and the associated detection methodologies vary substantially [34]. Resistance to one antibiotic can have several underlying resistance mechanisms and different detection methods will reveal different outcomes. Stewardson et al (2016) report the impact of resistance of *Enterobacteriaceae* to beta-lactams grouped as third-generation cephalosporin and found the additional LOS was 4.89 days [24], but when the exposure was limited to only ESBL-producing *Enterobacteriaceae*, the additional LOS increased to 6.8 days [25]. Hence, validation of methodologies and selection of appropriate cut-off thresholds need to be clearly reported and be consistent to ensure estimates remain comparable between studies.

Once exposure is standardised and clearly defined, economic studies need to consider the independent effect of the infection on the outcome variables. This would require adjusting for variables such as patient characteristics, underlying severity of disease [35], antibiotic therapy [36] and most importantly the timing of infection [8]. Adjustment of disease severity can be particularly challenging as measurements can alter the relationship between appropriate antibiotic therapy and outcome measures (i.e.; mortality) [37]. The optimal time of 24 h prior to culture collection represents the closest measure of the onset of infection and the most likely baseline measure of severity of illness [37]. Measurements taken at later times are likely to be on the causal pathway between exposure and outcome and should not be controlled for.

By far the greatest independent effect on hospital cost of AMR is LOS attributable to infection which is strongly dependent on time of infection and studies examining the healthcare costs of AMR should treat patient’s infection status as a time-dependent, rather than a time-fixed variable, to obtain more accurate LOS estimates [15]. In unadjusted analysis, this misclassification (i.e. time-dependent bias) can inflate LOS by as much as 9.8 days [15]. Multi-state survival modelling is a useful method that considers infection as an intermediate variable or a ‘state’ allowing individuals to move from one state to another when intermediary events occur, instead of assigning individuals to a fixed exposure on study enrolment [38]. In our systematic review, we only identified two studies that used multi-state modelling to fully adjust for the time-varying nature of infection and consider this the recommended method which would generate the most unbiased estimates of cost of AMR [24, 25]. This would suggest the large majority of current costing studies are generating longer LOS estimates leading to an inflated estimate of the cost of AMR.

Multi-state models can also be used to adjust for the impact of inappropriate empiric therapy on patient outcomes for the duration patients were receiving said therapy. Considering those with resistant infections are more likely to receive inappropriate empiric therapy, it is less severe source of bias in estimating the associated burden of AMR.

The key cost driver in healthcare studies is the valuation of a bed-day, often represented as an accounting cost but increasingly more common as an opportunity cost. Accounting costs indicate what has been spent to supply a bed, taking into consideration the total hospital budget [14]. In contrast, opportunity costs determine the value of achieving a desired outcome of freeing up a hospital bed for an alternative use [39, 40]. Our review identified one study that included both the accounting and opportunity costs for the same bug-drug combination, and provided readers with an alternative valuation of scarce healthcare resources [24].

The main limitation in this systematic review is the use of an untested quality grading tool. However, given the heterogeneity of included studies, it was agreed this was the most appropriate measure of quality assessment and it is based on identified factors that are important in evaluating economic studies [10]. However, it is possible that reviewers varied in how they used the tool, which may introduce unintended bias as to how the included studies were graded. The quality tool suggests aspects to consider about methodologies used to estimate costs of infection. It is important to consider the applicability of each aspect to the individual study under consideration. For example, time-dependent bias in extra LOS estimation can be eliminated by matching by day of infection. However, competing events bias still needs to be considered. Additionally, we did not distinguish whether studies required adjustment for each factor while using the tool. For example, we did not specify the classification of infections into healthcare-acquired and community-acquired infections, which would have determined whether adjusting for LOS prior to infection was necessary.

We specifically considered the incremental cost between a susceptible and a resistant infection. The bigger picture of costs of all infections and the need for infection prevention and control overall should not be ignored. Resistant infections may in fact need to be considered as additive [41], indicating that considering only the incremental cost of resistance compared to susceptible infection would underestimate the total costs of resistant infections.

## Conclusions

Rigorous and unbiased estimates of the economic burden of AMR are limited to healthcare-associated *Enterobacteriaceae* and MRSA BSIs. Valuation of economic cost of AMR for other infections and in other settings such as low or middle-income countries is particularly important given the high burden of disease and subsequent impact on health services. We make several recommendations to improve the quality of economic studies for generating high-quality estimates of the costs attributed to AMR. This is essential to inform decision makers around the globe not only about how to reduce the problem but also how best to allocate scarce healthcare resources.

## Additional files

Additional file 1: Systematic Review Protocol (DOCX 35 kb)

Additional file 2: Risk of bias tool used to assess methodological quality of included studies and criteria used to determine the quality of studies. (DOCX 19 kb)

## Abbreviations

€: Euro
AMR: Antimicrobial resistance
AUD: Australian dollar
BSI: Bloodstream infection
CCI: Charlson comorbidity index
CD: Canadian dollar
CHF: Confoederatio Helvetica Franc
CI: Confidence interval
*E. coli*: *Escherichia coli*
ESBL: Extended-spectrum beta-lactamases
INR: Indian rupee
*K. pneumoniae*: *Klebsiella pneumoniae*
LOS: Length of stay
MRSA: Methicillin-resistant *S. aureus*
*P. aeruginosa*: *Pseudomonas aeruginosa*
PRISMA: Preferred Reporting Items for Systematic Reviews and Meta-Analyses
*S. aureus*: *Staphylococcus aureus*
USD: United States dollar
VRE: Vancomycin-resistant *E. faecium*
