# Supplementary material for: Using the best available data to estimate the cost of antimicrobial resistance: a systematic review
Source: Antimicrob Resist Infect Control. 2019 Feb 1;8:26. doi: 10.1186/s13756-019-0472-z (PMC6359818; doi:10.1186/s13756-019-0472-z)
Supplement: Supplementary file 1 — Systematic Review Protocol (DOCX 35 kb) [file 13756_2019_472_MOESM1_ESM.docx]

**SYSTEMATIC REVIEW PROTOCOL**

**USING THE BEST AVAILABLE DATA TO ESTIMATE THE COST OF DRUG-RESISTANT INFECTIONS**

Authors: Teresa M Wozniak1,3, 5^*, Louise Barnsbee1,2,3^, Xing J. Lee 1,2,3Rosana E. Pacella4

1 Queensland University of Technology (QUT), Centre for Research Excellence in Reducing Healthcare Associated Infections, Brisbane Queensland, Australia.

2 Queensland University of Technology (QUT), Institute of Health and Biomedical Innovation, Brisbane Queensland, Australia.

3 Queensland University of Technology (QUT), Australian Centre for Health Services Innovation, Brisbane Queensland, Australia

4 University of Chichester, West Sussex, UK

5Menzies School of Health Research, Darwin Northern Territory, Australia

Corresponding author and guarantor:

Email: teresa.wozniak@menzies.edu.au

Address: 60 Musk Ave Kelvin Grove QLD 405

Author contacts

Teresa M. Wozniak, PhD [teresa.wozniak@menzies.edu.au](mailto:teresa.wozniak@menzies.edu.au)

Louise Barnsbee, BSc [louise.barnsbee@hdr.qut.edu.au](mailto:louise.barnsbee@hdr.qut.edu.au)

Xing J. Lee, PhD [xj.lee@qut.edu.au](mailto:xj.lee@qut.edu.au)

Rosana E. Pacella, PhD [r.pacella@chi.ac.uk](mailto:r.pacella@chi.ac.uk)

Update:

The protocol and subsequent review builds on an existing rapid review, but is not strictly designed to be an update of that review.

Amendment

This protocol is not an amendment of a previous protocol.

The protocol was reformatted on 10-OCT-2017 to incorporate revisions to the risk of bias tool.

Support

*Financial support.* TW and LB salaries are funded by the NHMRC-funded Centre for Research Excellence-Reducing Healthcare Associated Infections. Other than this salary support, the review did not receive sponsorship.

Rationale

Antimicrobial resistance is recognised as a threat to health on large scale due to the ability of resistant organisms to render antimicrobials obsolete. Sources provide various estimates of both the hospital LOS attributable to resistant compared to susceptible hospital acquired and community infections and the incremental economic costs of resistant versus susceptible infections.

Objectives

The objective of the review is to investigate the following questions:

Questions:

1. What are the methodologies of estimating the economic burden of drug resistant bacterial infections compared to drug susceptible?
2. What are the cost drivers for infections with a resistant organism compared to drug susceptible infections?
3. What are the costs attributable to drug resistant bacterial infections as compared to drug susceptible infections from both the health sector and societal perspectives.

The population to be studied includes adults with infection from any of the pre-specified organisms (see inclusion criteria). The exposure of interest is infection from antimicrobial resistant organisms and the comparator is infection from susceptible organisms. The outcomes of interest are excess LOS, mortality and cost of resistant compared to susceptible infection.

This protocol has been written in accordance with the PRISMA-P 2015 checklist (Shamseer et al., 2015).

Eligibility criteria

**Table 1. Inclusion and exclusion criteria for the systematic review.**

| Inclusions |
| --- |
| 1. Publications reporting empirical or primary evidence on the economic impact of resistant compared to susceptible infections using primary data collection, or publications reporting models of the impact of resistance. |
| 1. Publications reporting: Enterococcus, *Escherichia coli* (*E. coli*), *Klebsiella* *pneumonia* (*K. pneumoniae*), *Pseudomonas aeruginosa* (*P. aeruginosa*) or *Staphylococcus aureus* (*S. aureus*) (both community and healthcare acquired). |
| 1. Publications reporting the costs of resistant infections compared to susceptible infections. |
| 1. Publications reporting the control group as the susceptible strain of the organism (e.g. MRSA vs MSSA). |
| 1. Publications from year 2012 until end of searching (11th October 2016) |
| 1. Adult populations, defined as anyone admitted or presented to an adult hospital. |
| Exclusions |
| 1. Cost-effectiveness studies of interventions or studies focussed only on control interventions, research on antiviral, antimalarial or antiprotozoal. |
| 1. Research regarding reduced susceptibility to drugs rather than resistance to drugs |
| 1. Studies reporting resistant compared to uninfected controls (e.g. MRSA vs uninfected controls) |
| 1. Studies reporting only length of stay or only mortality. |

Information sources

Databases planned to be searched include Pubmed, Embase, Cinahl (through Ebscohost) and the Cochrane library. In the project planning stages it was identified that the excess costs of resistant infections from the societal perspective were found in reports published through the websites of reputable bodies such as the Review on Antimicrobial Resistance. Thus websites from reputable bodies were also searched for reports; further to the Review on Antimicrobial Resistance this included the RAND Corporation and London School of Hygiene and Tropical Medicine websites. Further reports were identified and suggested by experts.

Search strategy

In the project planning stage an initial search of the PubMed database was undertaken to identify relevant search terms by examining the titles and keywords of relevant articles. Overall, the aim was to search for articles which combined hospital costs, length of stay and/or mortality with antimicrobial resistant organisms.

Search strategies below:

| **Database** | **Strategy** |
| --- | --- |
| PubMed | ((((((("Health Care Costs"[Mesh]) OR "Cost of Illness"[Mesh]) OR "Hospital Costs"[Mesh]) OR "Episode of Care"[Mesh] OR "Length of stay"[Mesh]) OR "Costs and Cost Analysis"[Mesh] OR cost*[Title] OR economic*[Title] OR mortality[Title]) AND ("2000/01/01"[PDat] : "3000/12/31"[PDat]) AND English[lang])) AND (((((((((((((((((((((((((resistant infection*[Title]) OR antimicrobial resist*[Title]) OR antibiotic resist*[Title]) OR multi-drug resist*[Title]) OR multidrug resist*[Title]) OR Enterococcus faecalis[Title]) OR enterococc*[Title]) OR enterococcus faecium[Title]) OR Gram-positive bacterial infect*[Title]) OR Vancomycin resist*[Title]) OR "VRE"[Title]) OR "Escherichia coli"[Title]) OR e.coli[Title] OR "escherichia coli infect*"[Title ]) OR enterobacteriaceae[Title]) OR "Klebsiella pneumoniae"[Title]) OR "klebsiella infection*"[ Title]) OR "klebsiella pneumonia"[Title]) OR "pseudomonas aeruginosa" [Title]) OR pseudomonas[Title]) "pseudomonas infect*"[Title]) OR p.aeruginosa[Title] OR OR "MRSA"[Title] OR staphylococc*[Title]) OR "methicillin-resistant staphylococcus aureus"[Title]) OR "staphylococcus aureus"[Title]) OR methicillin-resist*[Title ] OR “Bloodstream infection”[Title] OR bacteremia[Title] OR bacteraemia[Title])) NOT "Tuberculosis"[Mesh]) NOT "Latent Tuberculosis"[Mesh]) NOT "Extensively Drug-Resistant Tuberculosis"[Mesh]) NOT "Tuberculosis, Multidrug-Resistant"[Mesh]) NOT Gonorrh*) NOT "Chlamydia"[Mesh]) NOT "Chlamydia trachomatis"[Mesh]) NOT "Chlamydia Infections"[Mesh] NOT "cost-effectiveness"  Filters activated: Publication date from 2012/01/01, Humans, English. |
| Embase | **#205 – limited to embase (excluded medline), limited to 2012, excluded tuberculosis and HIV and limited to english** |
| Ebscohost | ((TI "antibiotic resist*" OR TI "antimicrobial resist*" OR TI "multidrug resist*" OR TI "multi-drug resist*" OR TI "mrsa" OR TI "methicillin-resistant staphylococcus aureus" OR TI "staphylococc*" OR TI enterococcus OR TI "enterococcus faecalis" OR TI “enterococcus faecium” OR TI "gram-positive bacterial infection*" OR TI "vancomycin resist*" OR TI "VRE" OR TI "escherichia coli" OR TI "e.coli" OR TI enterobacteriaceae OR TI "klebsiella pneumoniae" OR TI "Klebsiella pneumonia" OR TI "Klebsiella infection" OR TI "p. aeruginosa" OR TI "pseudomonas" OR TI "pseudomonas aeruginosa" OR TI "bacteraemia" OR TI "bacteremia" OR TI "Bloodstream infection*") AND (((TI cost analysis) OR (TI cost*) OR (MW healthcare costs) OR (TI economic*) OR (TI cost-of-illness studies) OR (TI hospital costs) OR TI "Length of stay" OR TI "mortality" OR (TI episode of care)) |
| Cochrane | "healthcare cost*":ti or "Health care cost":ti or "cost of illness":ti or "cost-of-illness":ti or "hospital cost*":ti (Word variations have been searched) or "episode of care":ti or "length of stay":ti or "cost analyses":ti or cost:ti or cost:ti (Word variations have been searched) or economic:ti or "mortality":ti (Word variations have been searched) AND "resistant infect*":ti or "antimicrobial resist*":ti or "antibiotic resist*":ti or "multidrug resist*":ti or "multi-drug resist*":ti (Word variations have been searched) "enterococcus faecalis":ti or Enterococc*:ti or "entertococcus faecium":ti or "gram positive bacterial infection*":ti or "vancomycin resist*":ti (Word variations have been searched) or "VRE":ti or "Escherichia coli":ti or e.coli:ti or "Enterobacteriaceae":ti or "klebsiella pneumoniae":ti (Word variations have been searched) or "escherichia coli infect*":ti or "klebsiella infection":ti or "klebsiella pneumonia":ti or "pseudomonas aeruginosa":ti or "pseudomonas":ti (Word variations have been searched) or "pseudomonas infect*":ti or p.aeruginosa:ti or "MRSA":ti or staphylococc*:ti or "methicillin-resistant staphylococcus aureus":ti (Word variations have been searched) or "bloodstream infection":ti or "bacteremia":ti or "bacteraemia":ti (Word variations have been searched) |

The “*”symbol will be used where appropriate to include plural spellings in the search.

**Table 2: Draft of terms considered for searching.**

| Concept | MeSH or subject heading |
| --- | --- |
| Infection | Bloodstream infection*  Urinary Tract infection*  Urinary tract infection/economics/epidemiology/microbiology/mortality [MeSH]  Skin and soft tissue infection  Bacteremia/economics [MeSH] OR Bacteremia/epidemiology [MeSH] OR Bacteremia/microbiology [MeSH] OR Bacteraemia/mortality[MeSH  Sepsis |
| Antimicrobial | exp Anti-Bacterial Agents/ OR  exp Drug Resistance, Bacterial/ OR  exp Drug resistance, Microbial/ OR  antibiotic resistant.mp OR  *biotic* OR *microb*  Antibiotic resistance  Anti-Bacterial Agents/economics [Mesh]  Vancomycin/therapeutic use  Vancomycin/economics  Carbarpenum resistant antibiotics |
| Susceptibility | Antimicrobial-susceptible |
| Resistance | exp Drug resistance, multiple/ OR  Drug  Resistan*  MDR or  Multidrug-resistant (e.g. Multidrug resistant tuberculosis)  Multi-drug resistant microorganisms  Try multi-drug resistant/economics  Bacterial infections/economics/epidemiology/microbiology/mortality [Mesh]  Resistant infection*  Bacterial infections [mesh term]  Community-acquired infections/economics/ epidemiology/microbiology/mortality |
| Cost | exp “Cost and Cost analysis”/ OR  exp Cost of illness/ OR  exp Economic  [Mesh] Healthcare costs  Resource utilisation  Burden of isolation  Episode of care [Mesh]  Hospital charges  Length of stay [Mesh] |
| Limits | Year 2012 – current (October 2016)  English language only |
| NOT | Antiviral  Antimalarial  Antiprotozoal |

**Table3: Drafted search terms for specific organisms**

| Concept | Search Terms |
| --- | --- |
| *Enterococcus* |  |
|  | exp Enterococcus faecalis/ OR  exp Enterococcus/ OR  exp Enterococcus faecium/ OR  exp Gram-Positive Bacterial Infections/ OR  exp Vancomycin Resistance/ OR  VRE.mp. |
| *E.coli* |  |
|  | exp Escherichia coli/ OR  exp Escherichia coli Infections/ OR  Enterobacteriaceae OR  Escherichia coli.mp. |
| *K.pneumonia* |  |
|  | exp Klebsiella pneumoniae/ OR  exp Klebsiella Infections/ OR  Enterobacteriaceae OR  klebsiella pneumonia.mp. |
| *P.aeruginosa* |  |
|  | exp Pseudomonas aeruginosa/ OR  exp Pseudomonas/ OR  exp Pseudomonas Infections/ OR  pseudomonas.mp. |
| *S.aureus* |  |
|  | exp Staphylococcal Infections/ OR  exp Methicillin-Resistant Staphylococcus aureus/ or  exp Staphylococcus aureus/ OR  exp Gram-Positive Bacterial Infections/ OR  staphylococcus aureus.mp. |

Two investigators (TW and LB) had input into the search strategy and both independently scanned the titles, abstracts and then full text of appropriate articles. A third reviewer was available in the case of disagreement regarding articles included or excluded.

**Study records**

Data management:

Articles identified through database searching will be downloaded to and stored in Endnote. Data extracted from studies is planned to be recorded in a template.

Selection process:

TW and LB will screen articles by abstract, title and full text to assess articles against the inclusion and exclusion criteria.

Data Collection:

A data extraction template was designed, and LB and TW were each given a copy. Reviewers had the ability to add extra information to assist interpretation.

**Data Items**

The following data would be extracted from each study for inclusion:

1. Author, year, country, size of study population
2. Organisms included
3. Patient – community, ICU or surgery
4. Methodology

- Controlling for length of stay prior to infection- Y/N
- Adjustment for severity of illness – Y/N
- Adjustment for antibiotic therapy – Y/N

1. Economic perspective of study

- Hospital/healthcare - hospital morbidity, mortality, and cost
- Societal

1. Mortality, which may include:

- In-hospital only (all cause or disease specific)
- In-hospital and after discharge
- Attributable to infection

1. Morbidity

- Length of stay

1. Economic costs

- Hospital/healthcare costs (mean or median and the actual cost)
- Resource utilization

Outcomes and prioritization:

1. Length of stay (LOS) as reported by studies. This may include simple patient characteristics such as the total LOS of patients, or infection-related LOS of patients (e.g. days when infection was confirmed), or the results of models which investigate the incremental increase in LOS of resistant compared to susceptible infections.
2. Mortality of patients, as reported by studies.
3. Economic costs as reported by studies.

LOS is a common measure of morbidity helping to determine the amount of time patients stay in hospital. Length of stay attributable to resistant compared to susceptible infections is especially useful to determine the impact of resistant compared to susceptible infections. Length of stay is a driver of costs from an economic healthcare perspective, thus is an important consideration when considering the economic costs reported by studies.

Risk of bias (quality) assessment

We developed a risk of bias tool which addressed methodological limitations relevant to studies assessing the disease burden attributable to antibiotic-resistant infections, identified from literature (Gandra et al., 2014)

Data synthesis:

Data will be entered into a display table for discussion rather than statistically pooled or combined. No sensitivity or subgroup analysis is planned.

No meta-analysis will be conducted due to heterogeneity of studies.

Confidence in cumulative evidence:

As this review will consider important methodological considerations for studies assessing the disease and economic burden of resistant compared to susceptible infections, authors will discuss the merit of articles based on the specified methodological components. No GRADE approach is planned to be used.

References**:**

Gandra S, Barter DM, Laxminarayan R: **Economic burden of antibiotic resistance: how much do we really know?** *Clin Microbiol Infect* 2014, **20**(10):973-980.

Shamseer L, Moher D, Clarke M, Ghersi D, Liberati A, Petticrew M, Shekelle P, Stewart LA: **Preferred reporting items for systematic review and meta-analysis protocols (PRISMA-P) 2015: elaboration and explanation**. *BMJ (Clinical research ed)* 2015, **349**:g7647.
