# Supplementary material for: Using the best available data to estimate the cost of antimicrobial resistance: a systematic review
Source: Antimicrob Resist Infect Control. 2019 Feb 1;8:26. doi: 10.1186/s13756-019-0472-z (PMC6359818; doi:10.1186/s13756-019-0472-z)
Supplement: Supplementary file 2 — Risk of bias tool used to assess methodological quality of included studies and criteria used to determine the quality of studies. (DOCX 19 kb) [file 13756_2019_472_MOESM2_ESM.docx]

Risk of bias tool used to assess methodological quality of included studies and criteria used to determine the quality of studies.

| **Risk of Bias Item** | **Criteria for answers**  **YES (low to moderate risk of bias)** | **Criteria for answers**  **NO (high risk of bias)** |
| --- | --- | --- |
| 1. Adjustment for LOS prior to infection  Consideration of time-dependent bias | - Study has matched the resistant cases and susceptible control groups based on LOS prior to infection; or - Adjusted statistically for prior LOS; or - Sensitivity analysis has considered hospital LOS prior to infection. | - Study estimates excess LOS, mortality or costs without matching by LOS prior to onset of infection, or adjusting statistically for prior length of hospital stay; or - Expresses post-infection LOS; or - Expresses post-infection costs. |
|  | If multistate modelling was used to take into account the time-varying nature of antibiotic-resistant infections, studies were considered to have a low risk of time-dependent bias. | If multi-state modelling was not used or if a time-fixed analysis was conducted, studies were considered to have a high risk of time-dependent bias. |
| 2. Adjustment for comorbidities or severity of disease | - Study adjusts statistically for underlying co-morbidities on clinical outcomes; or - Study matches based on illness severity. | - Study estimates excess LOS, mortality or costs without matching for comorbidities or adjusting statistically for these ; or - Study does not adjust for severity of disease or comorbidities because prior univariate analysis determined no statistical significance between cases and controls for comorbidities measured. |
| 3. Adjustment for inappropriate antibiotic therapy | Study adjusts for inappropriate antibiotic therapy. | Study does not adjust for inappropriate antibiotic therapy. |

LOS: length of stay
